# Expression of genes associated with apoptosis in the residual yolk sac during the perihatch period of broiler chicks

**DOI:** 10.1016/j.psj.2022.101966

**Published:** 2022-05-21

**Authors:** Kaitlyn E. Reno, Sara E. Cloft, Eric A. Wong

**Affiliations:** Department of Animal and Poultry Sciences, Virginia Tech, Blacksburg, VA 24061, USA

**Keywords:** yolk sac, apoptosis, caspases, DNA fragmentation, broiler

## Abstract

The yolk sac (**YS**) consists of the yolk and the surrounding YS tissue, which provides essential nutrients and physiological functions for the developing embryo. After the YS is internalized into the abdominal cavity of the embryonic chick, the YS starts to degrade. Apoptosis, or programmed cell-death, is speculated to be the mechanism behind degradation of the YS. The objective of this study was to determine if degradation of the YS tissue was mediated by apoptosis during the perihatch period. The YS tissue was collected from broiler chicks from embryonic d 17 to d 7 posthatch. The mRNA abundance of genes that are involved in the regulation, initiation, and execution of apoptosis were analyzed by qPCR. The mRNA for Bcl2, Bcl2L11, cytochrome C and caspases 3, 6, 7, 8, 9, and 18 all showed a linear response from embryonic d 17 to d 7 posthatch. To confirm the role of apoptosis in the degradation of the YS tissue, a DNA fragmentation assay was performed. Degradation of genomic DNA in the YS tissue started on day of hatch. The characteristic ladder of oligonucleosomal-sized DNA fragments was observed on d 3, 5, and 7 posthatch. The combined gene expression and DNA fragmentation results demonstrate that degradation of the YS posthatch is mediated by apoptosis.

## INTRODUCTION

The yolk sac (**YS**) contains yolk and the surrounding YS tissue. The yolk provides essential nutrients for the developing chicken embryo, while the YS tissue provides essential metabolic functions and serves as the first line of defense against pathogens in the yolk (Wong and Uni, 2021). At around embryonic (**e**) d 19, the chick starts to internalize the YS in preparation for hatching and the YS starts to degrade (Romanoff, 1960). The residual internalized YS provides essential nutrition to both fed and delayed-fed chicks in the early period posthatch (reviewed in van der Wagt et al., 2020).

During midincubation, the YS tissue is responsible for the uptake of yolk contents and providing nutrition for the embryo (reviewed in Wong and Uni, 2021). Some yolk content is transferred to the intestines during late embryogenesis via the yolk stalk; however, Speake et al. (1998) reported that no yolk content was transferred to the intestines by the yolk stalk due to a tissue blockage. Van der Wagt et al. (2020) suggested that the discrepancy in findings about the yolk stalk might be due to the timing of the studies. It is possible that the degradation of the YS tissue during late embryogenesis removes the blockage or initiates the switch from yolk content absorption from the YS tissue to the yolk stalk.

Little is known about the mechanism of degradation of the YS. Apoptosis or programmed cell death has been proposed as the mechanism of degradation (Yadgary et al., 2014; Starck, 2021). Apoptosis is commonly seen across species during embryogenesis and plays a key role in regulating cell numbers in the intestine (Hall et al., 1994). Apoptosis is characterized by cell shrinkage, membrane blebbing, and DNA fragmentation that results in a distinct cellular morphology (Elmore, 2007). Unlike cell death by necrotic or pathogen-initiated destruction, apoptosis is controlled by a highly regulated series of events. The intrinsic or mitochondrial apoptotic pathway is one of the most common pathways for apoptotic cell destruction (Wang and Youle, 2009).

The Bcl2 protein family plays an important role in controlling the apoptosis pathway. Bcl2 is antiapoptotic and charged with protecting the mitochondrial membrane integrity (Cory and Adams, 2002). A member of the Bcl2 gene family, Bcl2L11 (Bcl2 like protein 11) is a proapoptotic protein that can inhibit Bcl2 and directly activate Bax and Bak proteins, which then activate caspases in humans and chickens (Luo and Rubinsztein, 2013; Cui et al., 2019). This pathway involves mitochondrial release of cytochrome C and activation of a group of caspases, which are cysteine proteases. In mammals, caspases are classified based on function as initiator caspases (caspase 8, 9, 10) or effector or executioner caspases (caspase 3, 6, 7). Slee et al. (1999) have determined the order of caspase activation during the cytochrome C initiated caspase cascade. The release of cytochrome C activates caspase 9, which then activates caspases 3 and 7. Caspase 3 activates caspases 2 and 6. Finally, caspase 6 activates caspases 8 and 10.

In the YS study by Yadgary et al. (2014), many genes associated with the cytoskeletal structure were downregulated from e17 to day of hatch. This study, however, only included samples up to day of hatch and did not include the days posthatch when the YS would most likely be undergoing apoptosis. Therefore, the objective of this study was to determine if apoptosis is involved in the degradation process of the YS from e17 to d 7 posthatch.

## MATERIALS AND METHODS

### Animal Husbandry and Sample Collection

Cobb 500 broiler eggs were obtained from a local hatchery and incubated at 37.5°C with 55% relative humidity. All animal procedures were approved by the Institutional Animal Care and Use Committee of Virginia Tech. On e17 and 19, day of hatch (**doh**), and d 1.5, 3, 5, and 7 posthatch, 7 embryos or chicks were randomly selected, humanely euthanized and used for collection of the YS tissue. For embryonic chicks, both the yolk-free BW and the weight of the YS were recorded. For posthatch chicks, BW and residual YS weight were recorded. The YS tissue was carefully removed from the yolk and rinsed in ice cold PBS. Samples were snap frozen in liquid nitrogen for later RNA and DNA extraction. The timepoints were picked based on the known timeline of YS internalization and degradation. The relative weight of the residual yolk was calculated by dividing residual yolk weight by chick BW and was used to merely track the weight change during the perihatch period and not to generate statistically significant data.

### RNA Extraction and Quantitative PCR

RNA was extracted from all 7 frozen YS samples at each time point by homogenizing tissue in TRI-Reagent and processing with the Direct-zol RNA Miniprep kit (Zymo Research, Irvine, CA). RNA concentration was determined with a Nanodrop 1000 spectrophotometer (Thermo Fisher Scientific, Waltham, MA). Complementary DNA was synthesized with 1 mg of total RNA and the High-Capacity cDNA Reverse Transcription kit (Applied Biosystems, Grand Island, NY) and analyzed using quantitative PCR (**qPCR**). The qPCR reactions consisted of 5 µL Fast SYBR green master mix (Applied Biosystems), 1 µL of forward primer (5 μM), 1 µL of reverse primer (5 μM), and 1.5 µL of diluted cDNA (1:30). All qPCR reactions were conducted in duplicate using an Applied Biosystems 7500 Fast Real-Time PCR system (Thermo Fisher Scientific). The cycling conditions were: 95°C for 20 s, followed by 40 cycles of 90°C for 3 s, and 60°C for 30 s. The primers used are listed in Table 1. GAPDH and RPLP1 were used as reference genes. The geometric mean of the Ct values for the 2 reference genes was subtracted from the Ct value of the target gene to obtain the ∆Ct value for each sample. The average ∆Ct value of the 7 e17 samples was used as the calibrator to calculate the ∆∆Ct and the fold change using the 2^−∆∆Ct^ method (Livak and Schmittgen, 2001).

**Table 1:** Primers used for qPCR.

Gene name	Function	Forward primer (5’ → 3’)	Reverse primer (5’ → 3’)	Amplicon size (bp)	Gene accession number
Bcl-2 B-cell lymphoma 2	Inhibits apoptosis	TCGTCGCCTTCTTCGAGTTC	CATCCCATCCTCCGTTGTCC	150	NM_205339.2
Bcl2L11 Bcl-2-like protein 11	Induces apoptosis	AGGCCGTCAGCCACTACCT	TCTTCTGCAAGCGAGTGAGATC	64	XM_025148907.2
Caspase 3	Effector caspase	AAAGATGGACCACGCTCAGG	TCCGGTATCTCGGTGGAAGT	189	NM_204725.1
Caspase 6	Effector caspase	TGCCAGATAGACGTGGGACT	AGTCATCCCGAGAGGCTTCA	141	NM_204726.1
Caspase 7	Effector caspase	TGGGTACACGCAATGGAACT	TCCTCACAGCTTCGGTCATT	103	XM_421764.5
Caspase 8	Initiator caspase	CTCCTACAGAAGCCCAAGCC	GGCATTTGCTTCCCTGCATT	162	NM_204592.3
Caspase 9	Initiator caspase	GGAACATTACGCCCGTTCTG	ACGATGTCTGACACCCGAAGT	60	XM_424580.6
Caspase 18	Present in chickens not mammals	GACCAGGCTGATGTTCTGGT	CCATCTTGTCACAGAGGCACT	106	NM_001044689.1
Cytochrome C	Initiate activation of caspase cascade	TTCCCAGTGCCATACGGTTG	GCTTGTCCTGTTTTGCGTCC	87	NM_001079478.1

### DNA Fragmentation Gel Electrophoresis Assay

DNA was extracted from 3 randomly selected samples from each time point using a modified version of the procedure of Herrmann et al. (1994). Briefly, 50 mg of YS tissue was added to 200 μL of 1x PBS, homogenized and centrifuged (5,000 x g for 10 min). The top yolk layer and supernatant were removed. The cell pellet was resuspended in 200 μL of 1x PBS. The protocol for DNA purification from blood using the Qiagen QIAamp DNA Blood Mini kit (Qiagen, Germantown, MD) was subsequently followed. The concentration of the DNA collected was determined with a Nanodrop 1000 spectrophotometer (Thermo Fisher Scientific). 1.5 μg of DNA were loaded per well of a 1.5% agarose/TAE gel containing 1x GelStar nucleic acid gel stain dye (Lonza, Rockland, ME). The Hi-Lo DNA marker (Minnesota Molecular, Minneapolis, MN) was used for estimating DNA fragment sizes. Gel images were captured using a ChemiDoc XRS+ Imaging System (Bio-Rad Laboratories Inc., Hercules, CA).

### Statistical Analysis

The fold change values for qPCR were logarithmically transformed to conform to normality. In a few instances for specific genes, some samples were omitted due to poor reproducibility or flagged as being an outlier. Outliers were removed from the dataset if they were beyond 1.5 times the interquartile range using a box and whisker plot by JMP v15 (SAS Institute, Inc., Cary, NC). Statistical analysis using least squares means contrasts was performed on the gene expression data to determine if there was a significant linear, quadratic, or cubic contrast to assess the relationship between gene expression and age of the chick during the perihatch period. In addition, a one-way ANOVA by day was conducted and where significant, a Tukey HSD test was used to separate the means. Statistical significance was considered *P* ≤ 0.05 in all instances.

## RESULTS AND DISCUSSION

### Relative Weight of Residual Yolk

**Figure 1:**
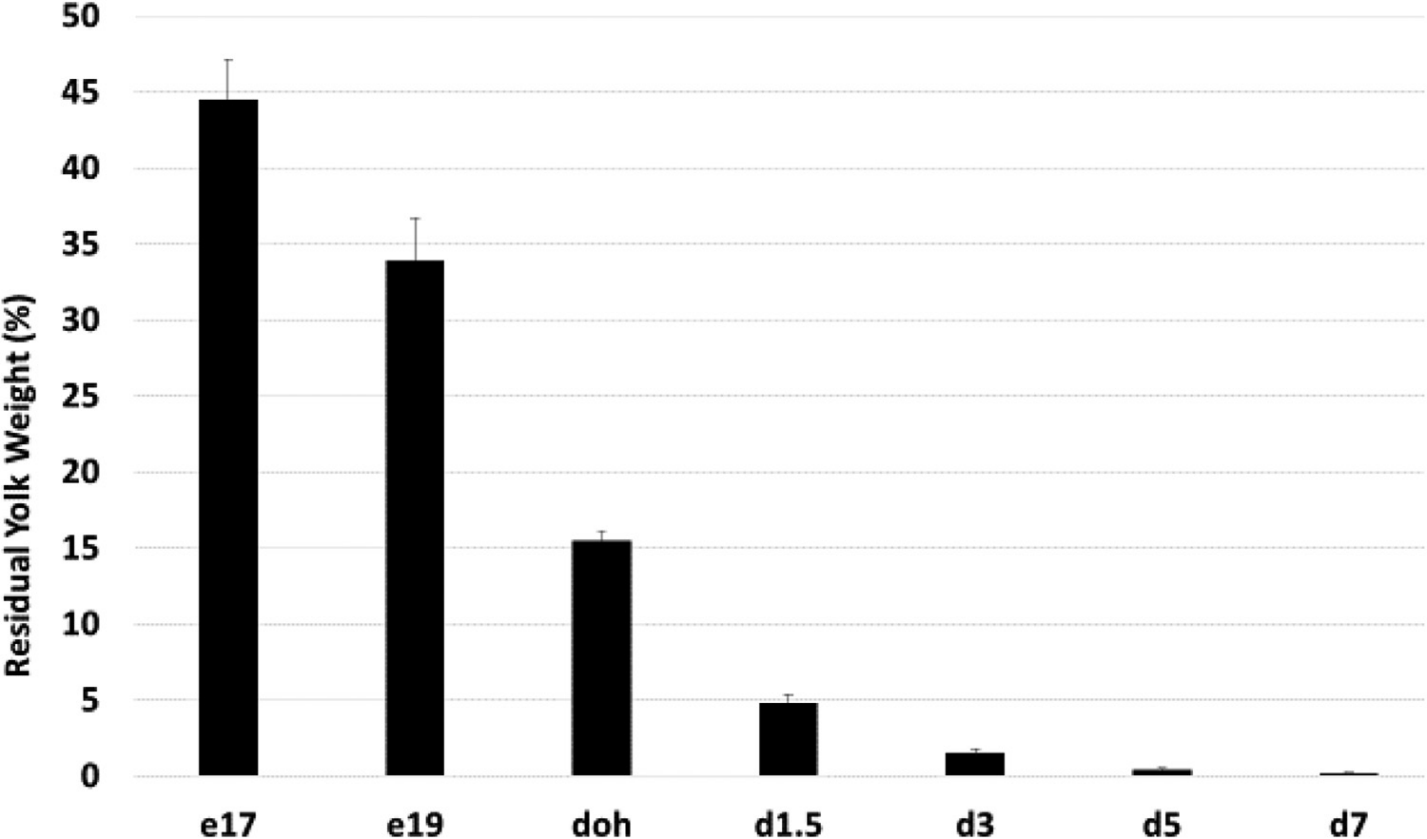
Relative weight of residual yolk during the perihatch period. Yolk weights were collected from broiler chicks (n = 7) at embryonic day (e) 17, and19, day of hatch (doh), and day (d) 3, 5, and 7 posthatch. Yolk weights were divided by yolk-free body weights to determine the relative weight of the residual yolk.

Relative weights of residual yolk decreased during the perihatch period (Figure 1). Relative yolk weights decreased from 45% at e17 to 34% at e19. After hatch, the weights of the residual yolk declined from 16% at doh to 0.2% at d7. The relative yolk weight at doh in our study was similar to the mean relative yolk weight of 12.8%, which was derived from the results of 126 published studies (Van der Wagt et al., 2020).

### Expression of Apoptosis Genes

**Figure 2:**
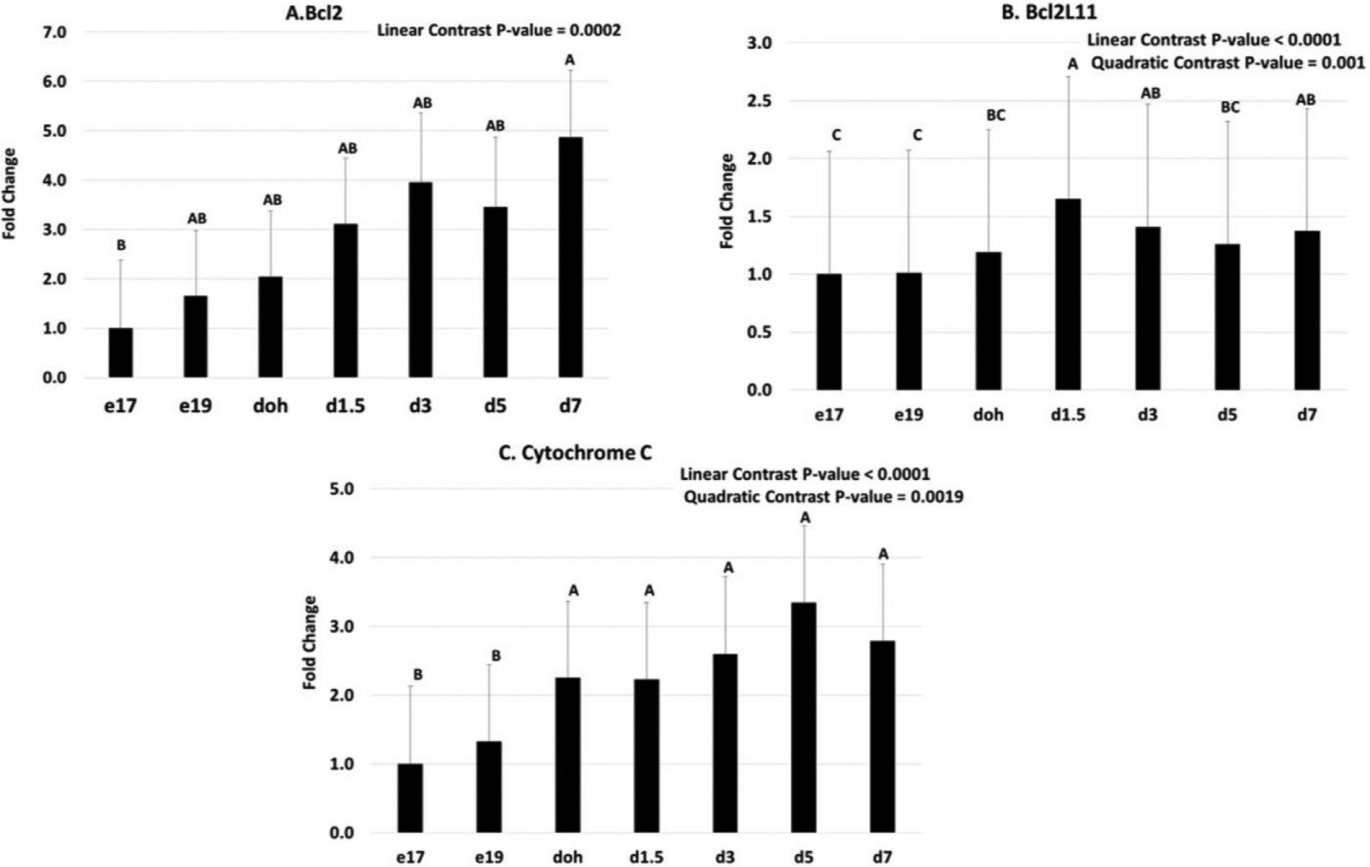
Temporal expression of genes regulating apoptosis in the yolk sac tissue. Yolk sac tissue was collected from broiler chicks at embryonic day (e) 17, and 19, day of hatch (doh), and day (d) 3, 5, and 7 posthatch. mRNA abundance for (A) B-cell lymphoma 2 (Bcl2), (B) Bcl-2-like protein 11 (Bcl2L11), and (C) Cytochrome C was determined by quantitative PCR. The e17 samples were used as the calibrator for calculating fold change. Linear, quadratic and cubic contrasts as well as a one-way ANOVA were conducted to assess the relationship between mRNA abundance and age of chick. Data presented are back transformed from logarithmic space. Bars with different letters are significantly different according to the Tukey HSD test (*P* ≤ 0.05; n = 5–7).

**Figure 3:**
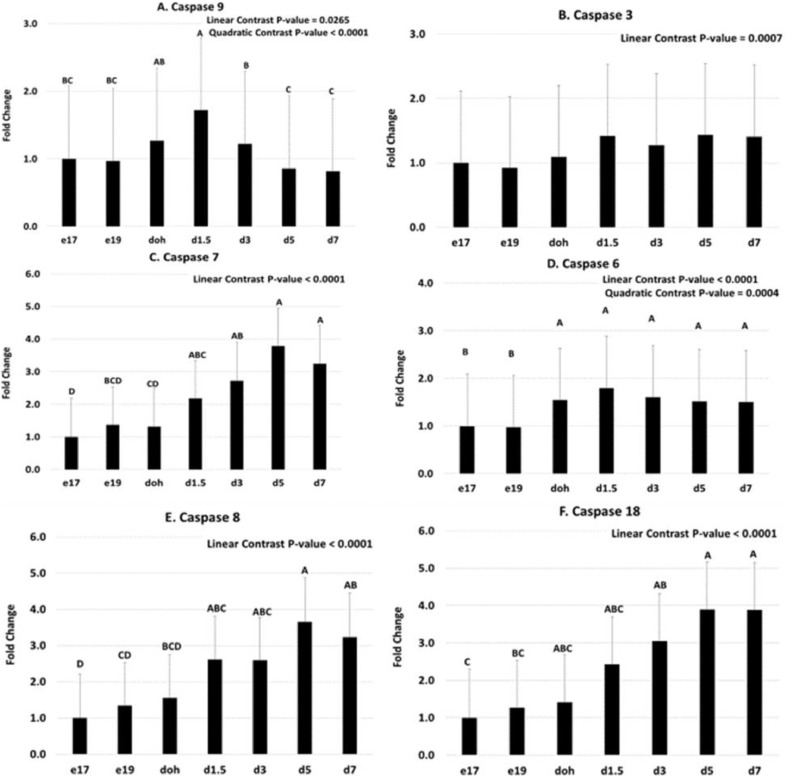
Temporal expression of caspase genes in the yolk sac tissue. Yolk sac tissue was collected from broiler chicks at embryonic (e) d 17 and 19, day of hatch (doh), and day (d) 3, 5, and 7 posthatch. mRNA abundance for (A) caspase 9, (B) caspase 3, (C) caspase 7, (D) caspase 6, (E) caspase 8, and (F) caspase 18 was determined by quantitative PCR. The caspase genes are presented in their proposed order of activation in the mammalian caspase pathway (Slee et al., 1999) rather than numerically. The e17 samples were used as the calibrator for calculating fold change. Linear, quadratic and cubic contrasts as well as a one-way ANOVA were conducted to assess the relationship between mRNA abundance and age of chick. Data presented are back transformed from logarithmic space. Bars with different letters are significantly different according to the Tukey HSD test (*P* ≤ 0.05; n = 6–7).

Apoptosis is mediated by pro- and antiapoptotic proteins that regulate the release of cytochrome C from the mitochondria, which in turn activate a cascade of caspases. The mRNA abundance of Bcl2, which is an anti-apoptosis protein, showed a linear increase from e17 to d7 (Figure 2A). The mRNA abundance of Bcl2L11, which is a proapoptosis protein and also known as BIM, showed linear and quadratic responses with an increase from e17 to d 1.5 followed by a decrease to d5 (Figure 2B). Cytochrome C mRNA showed both a linear and quadratic response, with lower levels during the embryonic period (e17 and e19) compared to the posthatch period (Figure 2C). All caspases showed lower mRNA abundance during the embryonic period compared to posthatch. Caspase 9 mRNA showed linear and quadratic responses, with an increase from e17 to d1.5 followed by a decrease to d5 and d7 (Figure 3A). Caspase 3 mRNA showed a linear increase from e17 to d7 (Figure 3B). Caspase 7 mRNA showed a linear response, with low expression from e17 to doh and greater expression at d5 and d7 (Figure 3C). Caspase 6 mRNA showed linear and quadratic responses, with an increase from the embryonic period (e17 and e19) to the posthatch period (doh to d7) (Figure 3D). Caspases 8 and 18 mRNA showed linear responses, with low abundance at e17 and e19 and greater abundance at d5 and d7 (Figures 3E, 3F).

In our study, the antiapoptotic Bcl2 and the pro-apoptotic Bcl2L11 both showed what appeared to be contradictory linear increases in mRNA from e17 to d7. Bcl2 is a prosurvival protein and works against the release of cytochrome C. Cory and Adams (2002), however, have speculated that Bcl2 indirectly regulates initiator caspases, which would perpetuate the apoptosis cascade once the cell has committed. This may explain why Bcl2 increases in our study in conjunction with Bcl2L11. The quadratic mRNA expression pattern for Bcl2L11 was consistent with its role as an initiator of apoptosis. Once apoptosis was initiated, expression of Bcl2L11 was no longer needed and therefore declined after d1.5

Apoptosis involves a cascade of events. Induction and upregulation of cytosolic cytochrome C production caused an accumulation of cytochrome C in the mitochondria (Chandra et al., 2002). As in our study, an increase in cytochrome C would lead to increased binding of cytochrome C with apoptotic peptidase activating factor 1 (Apaf-1) to form the apoptosome. The apoptosome would then activate the initiator caspase 9, resulting in activation of the downstream caspases (Shi, 2004).

The temporal pattern of expression of the caspases in the YS of the chick is consistent with the proposed order of action in the mammalian caspase cascade (Slee et al, 1999). In our study caspase 9 mRNA showed a quadratic expression pattern, like Bcl2L11, which is consistent with its role in initiating early events in the apoptosis pathway. Caspase 9 plays a role in the formation of the apoptosome prior to activation of downstream caspases, which all showed linear increases. Caspase 9 activates caspases 3 and 7 (Slee et al., 1999). Caspase 3 is the primary effector caspase and is important for nuclear condensation, DNA degradation and plasma membrane blebbing (Slee et al., 2001; Walsh et al., 2008). Caspase 3 is also required for the activation of caspase 6, which is an effector caspase that also serves to activate caspase 8 (Slee et al., 1999). In our study, caspases 7, 8, and 18 showed the latest increase in mRNA abundance, which is consistent with their downstream roles in the caspase cascade. Caspase 18 is present in the chicken, opossum, and platypus genomes, but is absent from the human and mouse genomes (Eckhart, 2008). The genomic structure of the chicken caspase 18 gene is most similar to chicken caspase 8, thus it is not surprising that the 2 genes showed a similar temporal pattern of mRNA expression. It is important to note that this analysis examined only mRNA levels and not protein levels or caspase activity.

The induction of genes in the apoptosis pathway in the YS, which degrades following internalization into the abdomen of the chick, is consistent with the genes involved in the mitochondrial apoptotic pathway in mammals (Wang and Youle, 2009). There is a transient induction of the proapoptotic protein Bcl2L11 and caspase 9, as well as an increase in cytochrome C. Induction of caspase 9 mRNA is followed by an increase in mRNA for the downstream caspases 3, 6, 7, 8, and 18. Induction of these caspases would lead to degradation of the cellular structures of the YS tissue, which would provide a source of nutrients for the developing chick.

### DNA Fragmentation Assay

**Figure 4:**
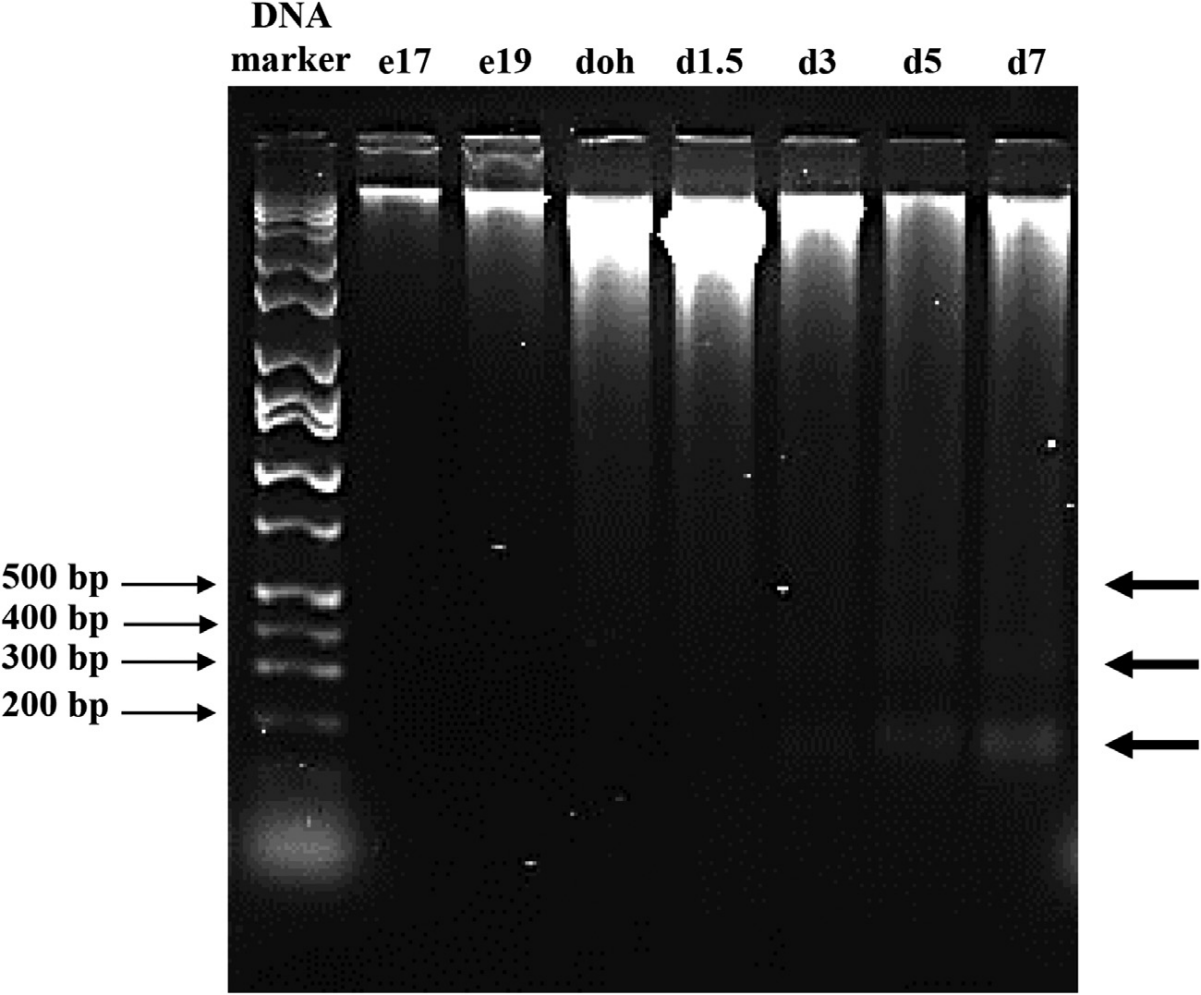
DNA fragmentation assay showing apoptosis in the yolk sac tissue. Yolk sac tissue was collected from broiler chicks at embryonic (e) d 17 and 19, day of hatch (doh), and day (d) 1.5, 3, 5, and 7 posthatch. Total genomic DNA was extracted from the yolk sac tissue and separated on a 1.5% agarose gel stained with GelStar. The DNA marker is a Hi-Lo DNA marker, with bands sizes in base pairs (bp) indicated by thin arrows. Thick arrows on the right side of the gel image indicate oligonucleosomal-sized DNA fragments characteristic of apoptosis.

A characteristic feature of apoptosis is DNA fragmentation of genomic DNA. An early step in apoptosis is the fragmentation of DNA into 20 to 300 kb sized fragments, which is followed by fragmentation into approximately 200 bp oligonucleosomal sized DNA fragments (Ioannou and Chen, 1996). In our study, at e17 and e19, genomic DNA consisted of predominantly high molecular weight DNA (Figure 4). At doh and d1.5, fragmentation of genomic DNA into large fragments was visible. The oligonucleosomal sized DNA fragments (∼200 bp) started to appear at d3 and became more prominent at d5 and d7. The DNA fragmentation coincided with the increase in expression of the caspases from d3 to d7.

### Summary

The YS is a multifunctional organ that provides nutrients and essential metabolic functions to the developing embryo, while its organs are developing and maturing. Around e17, the YS begins to degrade and the embryo prepares for hatching by internalization of the residual yolk. After hatch, the role of the YS diminishes and the YS begins to degrade. The degradation of the YS includes the upregulation of proapoptosis genes (Bcl2L11), cytochrome C, and a series of caspases. This increase in gene expression is accompanied by DNA fragmentation of genomic DNA. Together these results demonstrate that degradation of the YS during the perihatch period involves the apoptosis pathway.
